# Mathematical modelling of the oxytocin and vasopressin secretory system

**DOI:** 10.1016/j.coemr.2022.100341

**Published:** 2022-06

**Authors:** Duncan J. MacGregor

**Affiliations:** Centre for Discovery Brain Sciences, University of Edinburgh, Hugh Robson Building, George Square, Edinburgh, EH8 9XD, UK

**Keywords:** Oxytocin, Vasopressin, Secretion, Modelling, System

## Abstract

Magnocellular oxytocin and vasopressin neurons of the hypothalamus project to the posterior pituitary where they secrete their peptide hormone signals directly into the bloodstream. Their large anatomically distinct secretory mechanisms provide a uniquely accessible system in which to unite experimental and modelling approaches in the investigation of how input signals and electrophysiological properties of neurons relate to physiological function. We describe how the mechanisms have been translated and assembled into a mathematical model representation that can explain and simulate the complex and highly non-linear stimulus-secretion coupling of these neurons, and how this model has been applied to further understand these systems.

## Introduction

It is often forgotten that the output signal of most neurons is encoded, not in their action potentials or spiking, but in spike triggered secretion. Most commonly this is in synaptic transmission, where an axon conducted spike triggers Ca^2+^ entry and exocytosis of neurotransmitter containing vesicles, docked at the axonal terminal membrane. Secretion here is in small amounts and follows a rapid time course, making it very difficult to measure. The neuroendocrine cells of the hypothalamus use a similar mechanism of spike triggered exocytosis to secrete neuropeptides [1], but in larger quantities, and into spaces where they are subject to less rapid clearance. Neuroendocrine oxytocin and vasopressin neurons in particular, project directly to the posterior pituitary, where they secrete into blood plasma, forming a signal which is much more accessible to experimental measurement both *in vitro* and *in vivo*. This combined with the accessibility of these neurons to electrophysiology, where their cell bodies uniquely populate the supraoptic nucleus (SON) of the hypothalamus, makes them an excellent model for understanding stimulus-secretion coupling in neurons and other cells.

As well as peripheral secretion of oxytocin and vasopressin at the posterior pituitary, oxytocin and vasopressin neurons are known for dendritic secretion, generating autocrine, paracrine, and further signals, that act to modulate themselves, coordinate as a network with their neighbours, and signal other brain regions beyond. The focus here is on the modelling of the peripheral secretion signal, but there are likely to many common mechanisms and dynamics. Modelling of the vasopressin neurons’ distinctive phasic firing pattern and its interaction with the secretory mechanisms has been recently reviewed [2]. Dendritic secretion has been modelled in a simple form for its role in coordinating synchronised pulse generation in oxytocin neurons [3] in the milk ejection reflex.

## Data sources

**Figure 1:**
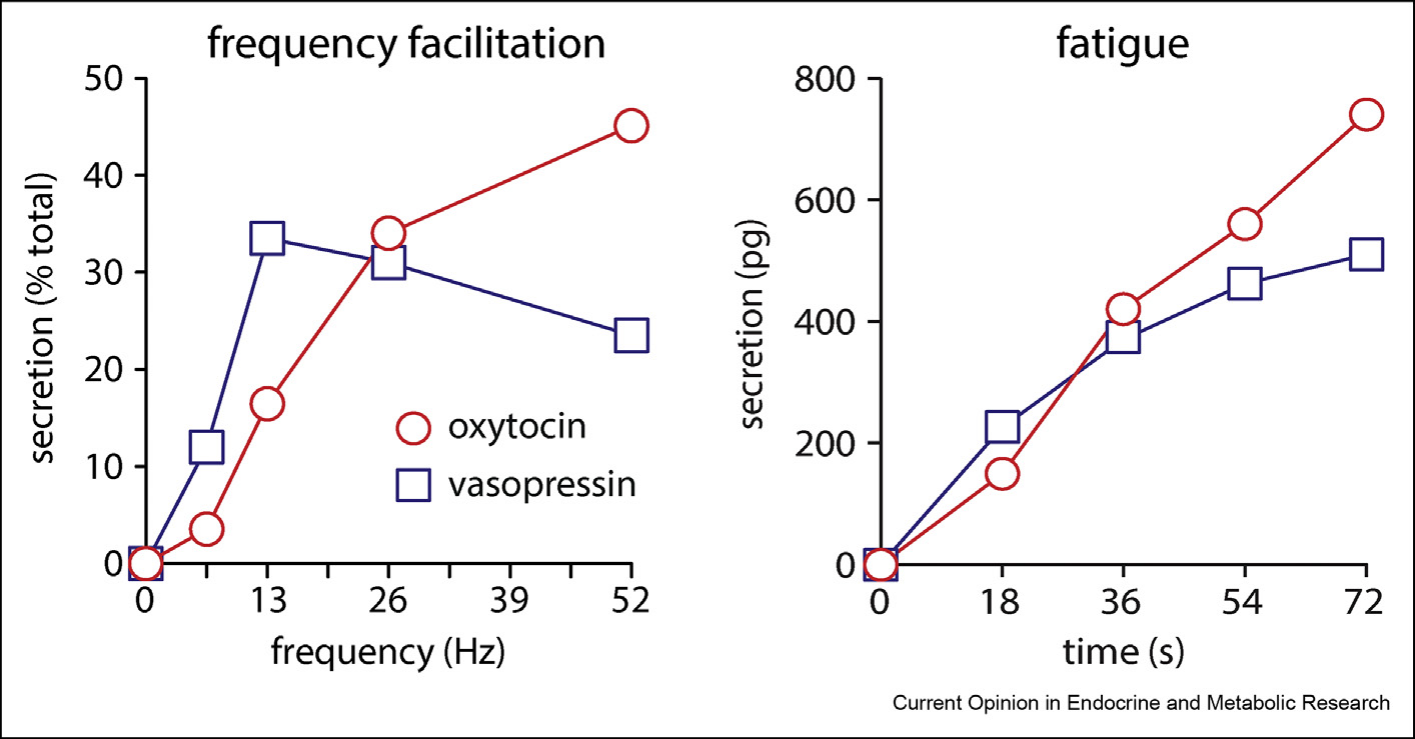
Frequency facilitation and fatigue in the stimulus-secretion coupling. Frequency dependence was investigated *in vitro* [7] by stimulating posterior pituitary glands with 156 pulses at four different frequencies and measuring the hormone secretion by radioimmunoassay. Facilitation peaks at 13Hz for vasopressin but continues to rise for oxytocin. Fatigue was measured [6] by stimulating at 13Hz for four different durations. Vasopressin shows a fall in secretion rate, while oxytocin shows no fatigue on this timescale.

The major source of quantitative data for the modelling is *in vitro* experiments performed in the 1970s and 80s [4, 5, 6, 7∗] where extracted posterior pituitary glands were used to measure secretion into a dish in response to electrical stimulation via an electrode, testing varied frequencies, durations, and patterns of electrical pulses intended to approximate the spikes conducted to the secretory terminals *in vivo*. Two major properties of the stimulus-secretion coupling were identified here: *frequency facilitation*, and *fatigue* (Figure 1). The amount of secretion is dependent not only on the number of spikes but also on their frequency, with the amount of secretion per spike increasing as the frequency increases. This facilitation effect varies between oxytocin and vasopressin. In oxytocin, secretion per spike continues to rise, although at a slowing rate, up to stimulation frequencies of 50 Hz. In vasopressin it peaks at around 13 Hz before gradually falling again.

Subject to prolonged stimulation over tens of seconds, the amount of secretion per spike, even at a fixed frequency, declines due to some mechanism of fatigue. Fatigue here refers to a time or use dependent reduction in the secretion response to a spiking stimulus. Again this varies, and is a much stronger effect in vasopressin than oxytocin. The combined effects of facilitation and fatigue result in a highly non-linear coupling between spiking and the rate of secretion, sensitive to both the rate and pattern of spiking activity. Similar properties of facilitation and fatigue have been detected in synaptic transmission [8]. This non-linearity is what makes considering secretion as the functional output signal important. If there was a simple linear relationship, of one unit of secretion per spike, then this distinction would be trivial. These dynamics make secretion sensitive to burst patterned spiking, which occurs in many types of neurons.

The data in these experiments gives a quantitative measure of the rate of secretion per spike, and how it changes. Modelling has two goals here; to explain these properties, based on knowledge of the underlying mechanisms, and to be able to simulate them, towards understanding their role in physiological function. Parallel work has developed robust integrate-and-fire based spiking models for vasopressin and oxytocin neurons [9,10]. Building a coupled model of spiking and secretion provides most of what we need to link the input and output signals of these neurons.

## Modelling and mechanisms

Modelling attempts focussed initially (RF Durie, PhD thesis, University of Edinburgh, 2008, https://era.ed.ac.uk/handle/1842/2220) on the dynamics of the secretory vesicle containing stores. Vesicles are synthesised and packaged in the cell body before being transported down the axon towards secretory terminals. At each terminal a small pool of vesicles is docked close to the secretory membrane ready for release via Ca^2+^ triggered exocytosis. These small pools, repeated over many thousands of secretory terminals, collectively form the *readily releasable* (or just *releasable*) *pool*. A second and much larger *reserve pool* is more abstract and less well anatomically defined, but accounts for the vesicles, which are in the vicinity of the releasable pools or being transported towards them. A simple model uses single variables to represent the content of each of these pools. The rate of secretion is a function of spiking activity, and the content of the releasable pool, depleting that pool. The releasable pool is refiled at some fixed, or activity dependent, rate from the reserve pool. This is capable of explaining fatigue if the rate of depletion exceeds the rate of refilling, depleting the releasable pool, and thus reducing the rate of secretion per spike until reduced demand allows refilling.

However, using reasonable parameters, based on estimates of the size of these pools, and the rate of secretion under stimulation, this mechanism is too slow to explain the relatively rapid fatigue observed in vasopressin neurons. Fatigue, and also facilitation, require some change in the coupling between spiking and the rate of vesicle exocytosis. Exocytosis is dependent on available vesicles, Ca^2+^ receptors, and Ca^2+^. Thus, the other important dynamics are in the relationship between spiking and Ca^2+^ entry. Spikes generated in the cell body are propagated down the axon through a process of regeneration. The spikes generated at the secretory terminal are subject to local excitability and ion channel properties, and can fail to propagate, or change in waveform. The best candidate mechanism for facilitation is frequency-dependent spike broadening [11]. Longer lasting, broader spikes produce more Ca^2+^ entry per spike, therefore increasing secretion per spike. Other known modulatory mechanisms, including Ca^2+^-dependent inactivation of Ca^2+^ channels [12], and a hyperpolarising Ca^2+^-activated K^+^ conductance [13], act to *reduce* Ca^2+^ entry per spike.

Ca^2+^ dynamics are highly complex, but can be simplified for modelling using compartments representing Ca^2+^ signals (and responses) with varied magnitude and time course parameters. These represent variations in the relationship between the sites of Ca^2+^ entry and receptors. If the receptors are close to the Ca^2+^ channels (as are the receptors that trigger exocytosis) then they experience a fast changing high magnitude Ca^2+^ signal. If the receptors are further away or more widely distributed, then they experience a lower magnitude but slower changing (longer lasting) intracellular Ca^2+^ signal.

## The working model

Another earlier modelling attempt [14], instead of basing on the underlying mechanisms, directly simulates the frequency response profile, and the rate of propagation failure, as a function of spike interval. It matches vasopressin concentrations in set conditions but lacks flexibility and a close quantitative match to the experimental data.

**Figure 2:**
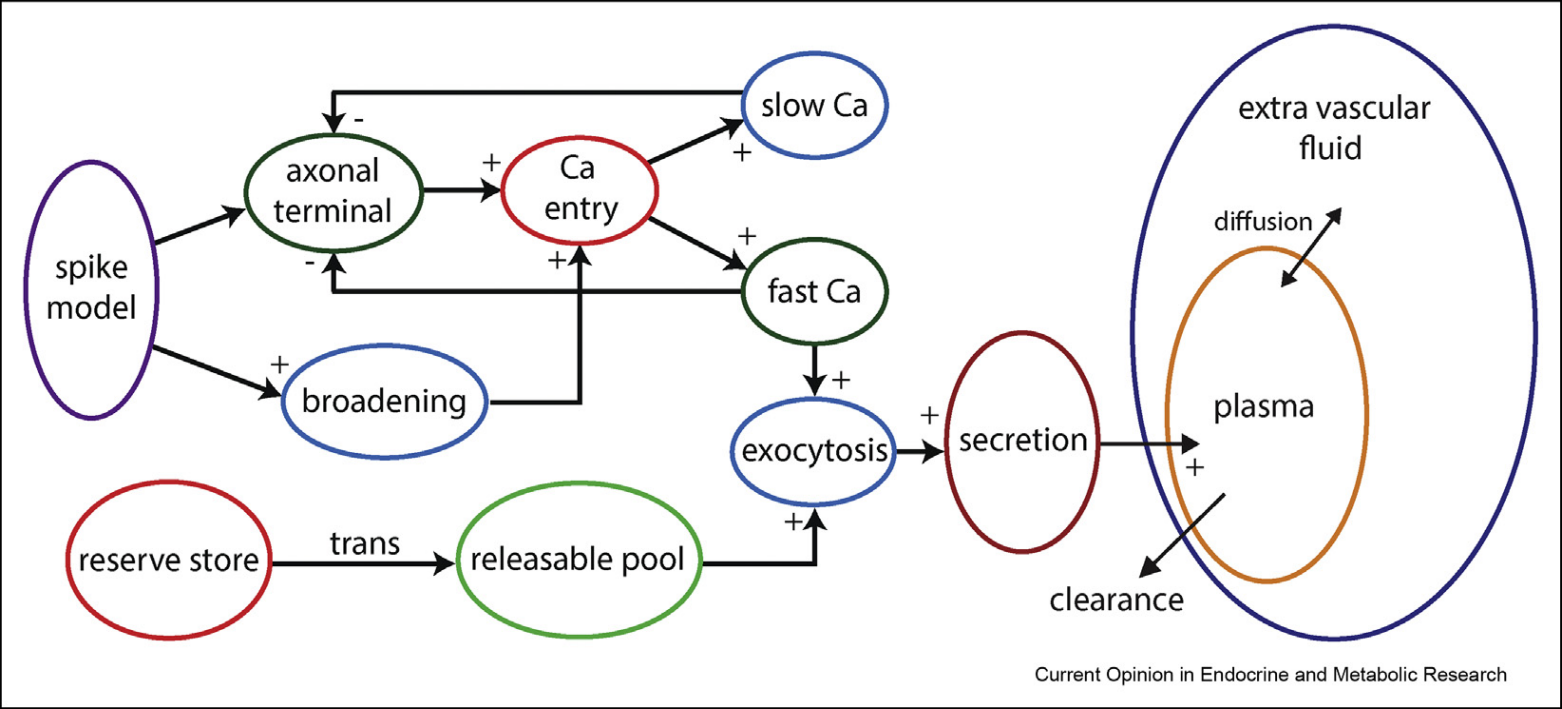
Coupled spiking, secretion, and plasma diffusion model. The secretion model [15], taking model generated spike events as an input, matches the secretion properties by combining representations of the vesicle pools and the competing excitatory and inhibitory Ca^2+^ dynamics at the secretory terminals. A few parameter changes, mainly reducing the Ca^2+^ dependent negative feedbacks, are sufficient to adapt between vasopressin and oxytocin secretion properties. The plasma diffusion model [17] accurately translates the secretion rate into plasma concentration, giving a strong quantitative match to experimental data.

The current working model [15] adds to the releasable and reserve pools of Durie's work a fast Ca^2+^ variable, a slow Ca^2+^ variable, and a variable representing spike broadening (Figure 2). The complex frequency dependence, observed especially in vasopressin neurons, peaking at ∼13Hz before falling again, is modelled by spike broadening driven facilitation of Ca^2+^ entry, opposed by a fast Ca^2+^ driven negative feedback control of Ca^2+^ entry. Any effects which directly track spike frequency must by nature be driven by fast changing signals. Ca^2+^ entry itself is represented by a factor in how each Ca^2+^ variable is incremented by a spike.

The rate of exocytosis is also driven by the fast Ca^2+^ variable. The Ca^2+^ binding and activation of exocytosis is thought to be cooperative [16] and the secretion rate factor, in the vasopressin model, uses the cube of the fast Ca^2+^ signal, which acts to match the initially highly non-linear relationship between spike frequency and secretion rate, particularly at low frequencies. The slow Ca^2+^ variable is used as a second negative feedback control on Ca^2+^ entry, representing an increased rate of spike propagation failure generated by the Ca^2+^-activated K^+^ conductance. This acts to model the relatively fast fatigue effect observed mainly in vasopressin neurons. Its action combines with depletion of the releasable pool, but dominates on shorter (tens of seconds) timescales.

These components, using five variables driven by differential equations [15], are sufficient to qualitatively and quantitatively match the observed frequency dependence and fatigue properties, and most importantly to match the resulting sensitivity of secretion to spike patterning as well as spike rate. The first interesting result, testing the role of the model's components, was that the ‘artificial fatigue’ generated by inhibiting spike transmission (the model's slow Ca^2+^ inhibition of Ca^2+^ entry), acts to resist depletion of the releasable store (i.e. real fatigue). The secretion rate is dependent on the releasable pool and thus protecting it maintains a more consistent signal response, purely dependent on the current input signal.

## Broader application and adaptation

The model was originally developed for vasopressin neurons. An interesting challenge to its robustness was adapting it to oxytocin neurons [17], which have very similar secretory mechanisms but show different frequency facilitation and fatigue properties. The oxytocin frequency response has a similar sensitivity to spike broadening, but appears to have less opposing inactivation of Ca^2+^ channels, resulting in a rate of secretion per spike which continues to increase up to very high spike rates. This likely corresponds to the high frequency spike activity observed in the oxytocin driven milk ejection reflex. Oxytocin neurons also show a much smaller fatigue effect, suggesting less Ca^2+^ driven hyperpolarisation of the secretory terminals and spike propagation failure.

In the model, these translate into reduced negative feedback control of Ca^2+^ entry by both the fast and slow Ca^2+^ variables. A less non-linear relationship between secretion rate and spike frequency, also suggests less cooperativity in the Ca^2+^ binding and activation of exocytosis, and so this is reduced to use the square instead of the cube of the fast Ca^2+^ variable. There is no specific experimental data available, but the modelling predicts differences in this mechanism [16] between vasopressin and oxytocin neurons. The parameters were precisely fitted by simulating the *in vitro* experiments that quantified these effects (Figure 1), testing varied frequencies and durations of spike stimulation.

A robust quantitatively fitted secretion model provides the opportunity to bridge experimental data, predicting plasma hormone concentrations from spike activity, and vice versa. The final element is a model of plasma diffusion and clearance, based on experiments that quantified these by infusing oxytocin, and measuring changing plasma oxytocin concentrations under various surgical interventions [18,19]. The model uses two compartments, for the extravascular fluid (EVF) and plasma. The hormone is secreted into the plasma, from where it acts and is cleared, but also diffuses, dependent on concentration gradient, between the plasma and EVF, slowing the rate of change of the plasma signal. The model uses the experimental data's accurate measures of the volumes, and diffusion and clearance rates, to translate the secretion model's rate output into experimentally accessible plasma concentration.

The integrated oxytocin spiking, secretion, and plasma model was first tested, and calibrated, matching the plasma response to injections of cholecystokinin (CCK) [17], a gut secreted signal which produces an excitatory spiking response in oxytocin neurons and a rapid elevation of oxytocin plasma concentration. The proper test of its quantitative fit was using different experimental data measuring the oxytocin plasma response to an excitatory osmotic stimulus, attempting to match this with no change to the model. This required a new model of how the osmotic input signal and fluid volumes respond to intra-venous or intra-peritoneal injection of saline, but it was able to closely match the output plasma signal, and develop understanding of how the plasma volume and osmo-sensitive components of the input signals interact [20].

The integrated input signal, spiking, secretion, and plasma models have already been extended to model populations of neurons, demonstrating the functional and physiological benefits of population heterogeneity [21]. Ongoing work is attempting to understand how the populations are coordinated to maintain long term function, and subject to plasticity to fulfil multiple roles. The secretion model is the vital part in being able to bridge experimentally accessible data, linking physiological input and output signals. With new techniques making detailed Ca^2+^ and secretion data more accessible, it can hopefully be applied to other neuroendocrine cells, and beyond.

## Conflict of interest statement

Nothing declared.
